# Network analysis of ABA-dependent and ABA-independent drought responsive genes in *Arabidopsis thaliana*

**DOI:** 10.1590/1678-4685-GMB-2017-0229

**Published:** 2018-07-23

**Authors:** Shiwei Liu, Zongyou Lv, Yihui Liu, Ling Li, Lida Zhang

**Affiliations:** 1 Department of Plant Science, School of Agriculture and Biology, Shanghai Jiao Tong University, Shanghai, China; 2 Department of Plant Science, School of Agriculture and Biology, Shanghai Jiao Tong University, Shanghai, China

**Keywords:** Drought stress, abscisic acid, RNA-seq, gene expression, protein-protein interaction network, Arabidopsis thaliana

## Abstract

Drought is one of the most severe abiotic factors restricting plant growth and yield. Numerous genes functioning in drought response are regulated by abscisic acid (ABA) dependent and independent pathways, but knowledge of interplay between the two pathways is still limited. Here, we integrated transcriptome sequencing and network analyses to explore interplays between ABA-dependent and ABA-independent pathways responding to drought stress in *Arabidopsis thaliana*. We identified 211 ABA-dependent differentially expressed genes (DEGs) and 1,118 ABA-independent DEGs under drought stress. Functional analysis showed that ABA-dependent DEGs were significantly enriched in expected biological processes in response to water deprivation and ABA stimulus, while ABA-independent DEGs were preferentially enriched in response to jasmonic acid (JA), salicylic acid (SA) and gibberellin (GA) stimuli. We found significantly enriched interactions between ABA-dependent and ABA-independent pathways with 94 genes acting as core interacting components by combining network analyses. A link between ABA and JA signaling mediated through a direct interaction of the ABA responsive elements-binding factor ABF3 with the basic helix-loop-helix transcription factor MYC2 was validated by yeast two-hybrid and bimolecular fluorescence complementation (BiFC) assays. Our study provides a systematic view of the interplay between ABA-dependent and ABA-independent pathways in response to drought stress.

## Introduction

Drought is one of the most severe abiotic factors restricting plant growth and yield (Xiong *et al.*, 2006). It has many different effects on plants and leads to a series of morphological, physiological, biochemical, and molecular changes that help optimize their growth (Farooq *et al.*, 2009). Abscisic acid (ABA) is a plant hormone that plays an important role in drought response. According to the response to ABA, drought responsive genes can be broadly classified into two groups: ABA-dependent and ABA-independent genes (Yamaguchi-Shinozaki and Shinozaki, 2005). A *cis*-element analysis has revealed that many ABA-dependent drought-related genes contain a conserved ABA-responsive element (ABRE) with ACGT cores in their promoter regions (Uno *et al.*, 2000). The expression of these genes is mainly regulated by the basic-domain leucine zipper (bZIP), including ABRE-binding proteins (AREB) or ABRE-binding factors (ABFs) (Uno *et al.*, 2000; Fujita *et al.*, 2009). Among the AREB/ABF subfamily of basic leucine zipper (bZIP) transcription factors in the Arabidopsis genome, *AREB1/ABF2*, *AREB2/ABF4* and *ABF3* are induced by both drought stress and ABA (Fujita *et al.*, 2013). Over-expression studies have shown that these three AREB/ABFs are positive regulators of ABA signaling under drought stress (Kang 2002; Kim *et al.*, 2004; Furihata *et al.*, 2006; Fujita *et al.*, 2009). These studies indicated these three AREB/ABFs as core transcription factors that cooperatively regulate ABRE-dependent gene expression in ABA signaling during response to drought stress.

Although several drought-responsive genes are involved in the ABA signaling pathway, many drought-induced genes do not respond to ABA treatment, suggesting the existence of ABA-independent pathways in response to drought stress (Shinozaki 2000). The regulation of these ABA-independent genes occurs through the drought responsible element (DRE) and C-repeat (CRT) *cis*-acting elements, in combination with DRE-binding protein (DREB) or C-repeat-binding factor (CBF) transcription factors. These have an APETALA2 (AP2) DNA-binding domain, such as DREB2, playing a pivotal role in ABA-independent gene expression under drought stress (Shinozaki 2000; Tran *et al.*, 2004; Sakuma *et al.*, 2006). Some other transcription factors such as MYB/MYC and WRKY in ABA-independent signaling have been demonstrated to be involved in regulating the response to drought stress in plants (Abe *et al.*, 1997). Additionally, NAC transcription factors are also well known to play important roles in both ABA-dependent and ABA-independent signaling under drought stress (Hu *et al.*, 2006).

Recent studies have revealed that the relationships between ABA-dependent and ABA-independent signaling pathways are much more complex (Nakashima *et al.*, 2009; Nakashima *et al.*, 2014; Roychoudhury *et al.*, 2013). For example, instead of simple relationships between ABA-dependent and ABA-independent signaling pathways, recent studies have raised the intriguing possibility of crosstalk between these two major type pathways (Yoshida *et al.*, 2015). As core components in ABA signaling, the three subclass III SNF1-related protein kinases 2 (SnRK2s) likely participate in the convergence of ABA-dependent and ABA-independent pathways, regulating the expression of *AREB/ABFs* and *DREB* under drought stress (Fujita *et al.*, 2013). Moreover, recent studies have shown that the ABA-dependent proteins AREB1/ABF2, AREB2/ABF4 and ABF3 play important roles in the regulation of the drought response by interacting with the ABA-independent proteins DREB2A, DREB1A and DREB2C (Lee *et al.*, 2010; Kim *et al.*, 2011). The gene ANAC096, encoding a key NAC transcription factor in the ABA-independent signaling pathway, has been shown to physically interact with the ABA-dependent transcription factors ABF2 and ABF4 to regulate gene expression in response to drought stress (Xu *et al.*, 2013). These increasing molecular evidences show that there are the complex relationships between ABA-dependent and ABA-independent signaling pathways; however, how extensive the interplay is between two major signaling pathways during the response to drought stress remains unclear.

The development of high-throughput data-collection techniques, such as deep sequencing, provides a better understanding of gene expression on a global scale (Wang *et al.*, 2009). Increasing molecular information has prompted the development of gene networks through computational approaches for plants, thus providing an overview of gene-gene relationships at the system level (Cui *et al.*, 2008; Lin *et al.*, 2011; Zhang *et al.*, 2016; Liu *et al.*, 2017). To advance our understanding of the molecular mechanism underlying drought response, we combined RNA sequencing and network analysis to explore the highly possible interactions functioning in crosstalk between ABA-dependent and ABA-independent drought signaling pathways in Arabidopsis. Our report provides a systematic view of the interplay between ABA-dependent and ABA-independent pathways in response to drought stress. This method will facilitate the discovery of gene-gene relationships for studies of the molecular mechanism underlying the drought response in Arabidopsis and will provide a reference for crop plants.

### Material and Methods

#### Plant material and sample treatment

Columbia wild-type *Arabidopsis thaliana* seeds were sterilized with 10%NaClO solution and 0.1% TritonX-100 for 5 min before plating, and then washed 3 times with distilled water. Seeds were grown on Murashige-Skoog (MS) medium with 0.6% agar and 3% sucrose and kept for 3 days at 4 °C to break the dormancy. The medium was then placed in a growth chamber with a cycle of 16 hours of light and 8 hours of dark at 22°C. After 5 days, 2 true leaves of the each seedling grew out. The seedlings were transferred into pots with soil (vermiculite: peat: perlite = 7: 3: 1). The pots were placed in a chamber under a photoperiod of 16/8 h (light/dark) at 22 °C at a relative humidity of 35-60% (Han *et al.*, 2013; Jin *et al.*, 2013). One-week-old plants were drought stressed by withholding water for 10 and 12 days, and then 12-day drought stressed plants were rewatered for 2 days for recovery (Baker *et al.,* 1994; Jin *et al.*, 2013). Three-week-old plants grown under well-watered condition were sprayed with 10 μmol/L ABA (Sigma) and kept in light for 4 hours (Li *et al.*, 2001). All of the samples were frozen in liquid nitrogen for further analysis.

#### RNA extraction

RNA was extracted with the RNAprep pure Plant Kit purchased from Tiangen Biotech (China). Concentration and purity of the extracted RNA were tested using a Nanodrop 2000 spectrophotometer. RNA integrity was checked through agarose gel electrophoresis, and the RNA integrity number (RIN) values were measured by an Agilent 2100 analyzer. The results satisfied the requirement that the total RNA be more than 5 μg, the concentration more than 200 ng/μL, and the OD 260/280 between 1.8 and 2.2. The RNA samples from eight plants in each situation were then mixed for RNA sequencing.

#### Illumina sequencing and data preprocessing

cDNA libraries were constructed using an RNA sequencing assay for paired-end transcriptome sequencing. Library construction and sequencing were done by MajorBio Sequencing Company (China). Sequencing was done on an Illumina HiSeq 2500 instrument with 8-gigabase in-depth sequencing, which was used to obtain more detailed information on gene expression.

The sequencing reads were statistically analyzed and quality assessed by FASTQC (http://www.bioinformatics.babraham.ac.uk/projects/fastqc/) and then processed by Trimmomatic to remove adapter sequences and low-quality reads with average quality scores lower than 15 (Bolger *et al.*, 2014). Reads that were less than 50 base pairs (bp) after trimming were also excluded from further genome mapping.

#### Data access

The RNA-seq data of this study have been deposited in NCBI Sequence Read Archive (SRA, http://www.ncbi.nlm.nih.gov/Traces/sra) with accession number: SRP075287.

#### Analysis of differentially expressed genes

The cleaned reads were mapped to the Arabidopsis genome using TopHat2 (Trapnell *et al.*, 2012). Gene expression levels were expressed as fragments per kilobase of transcript per million mapped reads (FPKM). Differentially expressed genes (DEGs) were identified by a significance analysis using Cuffdiff with a*p*-value < 0.05 and at least two-fold change. Gene cluster analysis using Euclidean distance and complete linkage was performed and displayed with MultiExperiment Viewer (Howe *et al.*, 2011).

#### Functional enrichment analysis

The Gene Ontology (GO) enrichment analysis is based on the tool available in the DAVID database (Huang *et al.*, 2008). We identified significantly enriched GO terms of DEGs with FDR < 0.05.

#### Real-time qRT-PCR analysis

Approximately 500 ng of total RNA was used for cDNA synthesis using the PrimeScript^TM^ RT Reagent Kit and amplified using the SYBR Green Mix Kit (Takara) by quantitative real-time PCR on a Light Cycler 96 real-time PCR system (Roche Applied Science). Primers were designed using the Primer Premier software based on the gene sequences, and the length of amplified fragments was 100-250 bp (Table S1). PCR cycling conditions for amplification were 94 °C for 60 s, followed by 45 cycles of 94 °C for 20 s, 55 °C for 20 s, and 72 °C for 20 s. Relative expression levels were determined using the 2^-ΔΔCt^ analysis method (Livak *et al.*, 2001). Three biological replicates for each sample were used for real-time PCR analysis. The relative expression level of each target gene was normalized to the level of the reference ubiquitin *(AtUBQ)* gene (GenBank accession number: NM116771.5; Locus: AT4G05320).

#### Gene interaction analysis

We analyzed the drought-responsive genes involved in the interplay between ABA-dependent and ABA-inde-pendent pathways based on the global Arabidopsis protein-protein interaction (PPI) network (AraPPINet) (Zhang *et al.*, 2016). To estimate the significance of an ABA-dependent drought-related gene linked to the whole ABA-independent gene set, an edge-shuffling method was used to generate 1000 randomized networks with the same degree of each node as that in AraPPINet. The number of interactions between each of ABA-dependent drought-related genes and the whole ABA-independent gene set was then counted in AraPPINet and the randomized networks. The *p*-value was calculated by counting the proportion of values from the empirical distribution that were equal to or greater than the observed value of AraPPINet. Similarly, the significance of an ABA-independent drought-related gene linked to the whole ABA-dependent gene set was also estimated by this method. Genes were identified as core interacting components involved in the interplay between ABA-dependent and ABA-independent pathways with an empirical *p*-value < 0.05.

To estimate the statistical significance of global interactions between ABA-dependent and ABA-independent drought-related genes in AraPPINet, the Z-score transformation normalization method was used to compare interactions of the two gene sets and that of non-DEGs in AraPPINet. Z-scores were calculated by the following equation:

$$\text{Z}-\text{score}=\frac{{\text{N}}_{\text{AraPPINet}}\;-{\text{μ}}_{\text{randnet}}}{{\text{SD}}_{\text{randnet}}}$$

where N_AraPPINet_ is the number of interactions between drought-responsive genes in AraPPINet,μ_randnet_ is the average number of interactions of those genes in the randomized networks, and SD_randnet_ is the standard deviation in the randomized networks (Stelzl *et al.*, 2005). We calculated the significant enrichment of interactions between drought-responsive gene sets by comparing the Z-scores of DEGs with those of non-DEGs using t-tests. The predicted PPI network between drought-responsive genes was drawn using Cytoscape (Cline *et al.*, 2007).

#### Yeast two-hybrid assays

Gene *ABF3* cDNA (encoding full-length protein) was cloned into pGADT7 vector (AD-ABF3), and *MYC2* cDNA (encoding full-length protein) was cloned into vector pGBKT7 (BD-MYC2) using the ClonExpress MultiS One Step Cloning Kit (Vazyme). Plasmids were co-transformed into yeast strain AH109. The interaction between ABF3 and MYC2 was tested through monitoring the growth of the transformed colonies on SD double dropout (2D) plates without Leucine and Tryptophan (-LEU, -TRP) and SD quadruple dropout (4D) plates without Leucine, Tryptophan, Histidine and Adenine (-LEU, -TRP, -HIS, -ADE). The experiments were replicated three times, and 10 mM 3-Amino-1,2,4-triazole (3-AT) was added to inhibit the self-activation on 4D plates. The plates were grown at 30 °C.

#### Bimolecular fluorescence complementation analysis

All constructs for the Bimolecular Fluorescence Complementation (BiFC) assay were obtained by PCR amplification and cloning into vector pXY104 and vector pXY106, as described previously (Yu *et al.*, 2008). Vector pXY106 contains the N-terminal fragment of the yellow fluorescent protein (nYFP), and vector pXY104 contains the YFP C-terminal fragment of (cYFP). The constructs were co-transformed into tobacco (*Nicotiana benthamiana*) leaf epidermal cells through *Agrobacterium tumefaciens* strain GV3101 at the indicated combinations. After culturing for 48 hours, cells were observed and analyzed by confocal microscopy (Leica TCS SP5) with an argon laser. YFP was excited at 514 nm, and the emitted light was captured between 530-600 nm.

### Results

#### Identification of ABA-dependent and ABA-independent drought-responsive genes

Plants were grown under well-watered conditions, and drought stress was applied by withholding water for 10 and 12 days after sowing, respectively. In order to understand the global effects of drought stress on gene expression of ABA-dependent and ABA-independent pathways, RNA sequencing was used to profile gene expression levels under drought stress and ABA treatment with corresponding controls in samples. Illumina sequencing yielded more than 356 million reads for eight samples after removing low-quality reads, and 87.1–93.4% of the high-quality reads were mapped to the reference genome sequence, covering more than 22,000 genes in the Arabidopsis genome (Table 1). To evaluate whether most genes can be detected by RNA-sequencing in the study, we calculated the number of detected genes by subsampling the raw reads from each library (Tarazona *et al.*, 2011). The number of genes detected with at least five mapped reads approached saturation at roughly 5 million reads for all samples (Figure S1), and in fact, the majority of genes detected at all reads were already detected within the first 30% reads. These data showed that the sequencing depth would be sufficient to detect most of the expressed genes in Arabidopsis genome in this study.

**Table 1:** Statistical summary of RNA sequencing data.

Library	No. of raw data pairs	No. of clean read pairs of high quality	No. of clean mapped reads	Reads mapped at high quality (%)	No. of mapped genes
Drought-10d	31,980,648	27,079,386	50,584,293	93.4	22,730
Control-10d	31,806,266	27,184,358	47,355,152	87.1	22,277
Drought-12d	22,444,685	18,902,136	35,195,777	93.1	22,563
Control-12d	29,784,408	24,936,590	45,683,833	91.6	22,686
Rewatered-2d	28,918,056	23,747,737	43,885,818	92.4	22,675
Control-14d	31,806,685	25,846,352	46,730,204	90.4	22,637
ABA-4h	28,770,889	23,301,937	41,524,052	89.1	22,623
Control-4h	30,995,484	25,425,280	45,460,401	89.4	22,602

**Figure 1:**
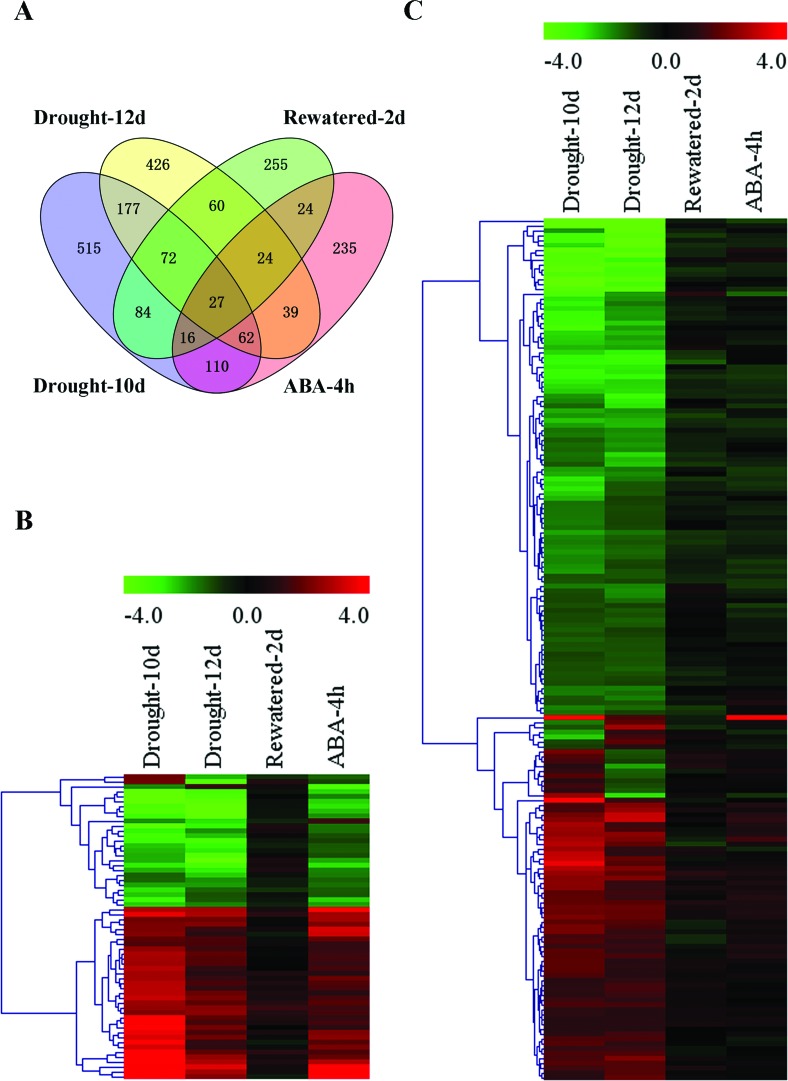
Venn Diagram and Clustered Heatmap of DEGs. (A) Venn diagram of DEGs. Venny (http://bioinfogp.cnb.csic.es/tools/venny/index.html) was used to compare DEG lists with Venn diagrams. (B) Clustered heatmap of ABA-dependent DEGs. (C) Clustered heatmap of ABA-independent DEGs. Each row in the heatmap represents a gene, and each column represents a treatment. Red and green indicate up-regulated and down-regulated expression, respectively. The Euclidean formula was used for distance calculation, and a complete linkage clustering analysis was performed on DEGs. The log_2_ ratios of the expression of DEGs in each sample were used for a cluster analysis.

All four experimental situations were analyzed to identify differentially expressed genes (DEGs) with a fold change > 2 and a *p*-value < 0.05. We identified a total of 1,612 genes that were significantly differentially expressed after 10 or 12 days of drought stress (Figure 1A). Among these DEGs, 211 genes were responsive to both drought stress and ABA treatment (Figure 1B), while 1,118 genes were specifically responsive to drought stress but whose expression levels were not obviously affected by exogenous ABA treatment (Figure 1C), indicating that more ABA-independent genes were involved in response to prolonged drought stress. The expression levels of the other 283 DEGs were not obviously recovered by rewatering, suggesting that these DEGs could not rapidly change their transcription in response to the switch from drought stress to well-watered conditions, or might not be involved in the drought response. More interestingly, the clustered heatmap indicated that most of the ABA-dependent DEGs showed a similar expression pattern under drought stress and ABA treatment (Figure 1B), suggesting that these genes were common elements shared by the drought stress signal and ABA in the signaling pathway.

#### Functional enrichment of ABA-dependent and ABA-independent drought-responsive genes

**Figure 2:**
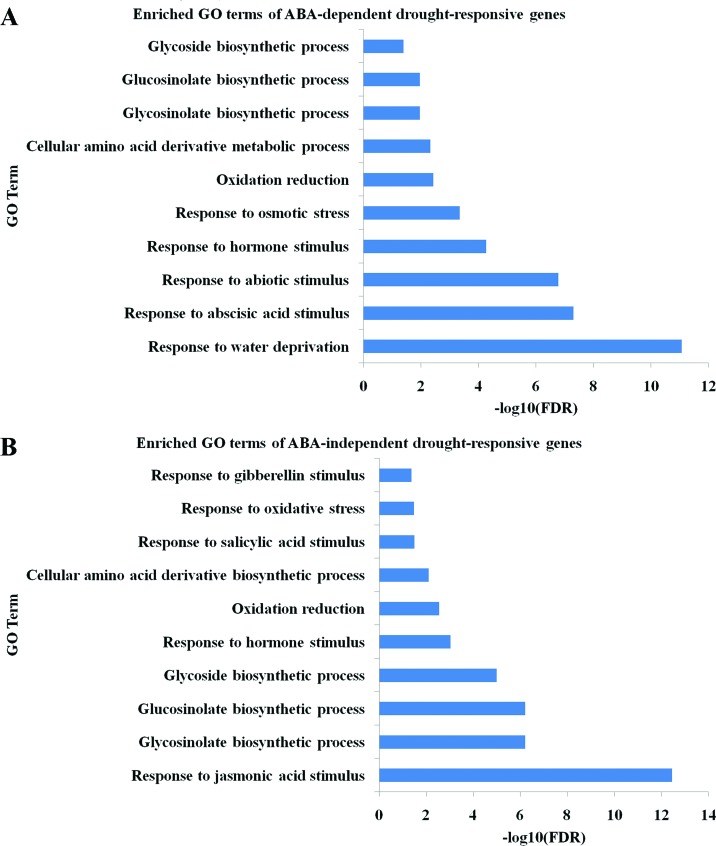
Functional enrichment of (A) ABA-dependent and (B) ABA-independent drought-responsive genes. The x-axis represents the negative log_10_(FDR) of enriched GO terms. The y-axis represents the GO terms.

To reduce the noise, the genes that were not obviously recovered by rewatering were discarded in further analyses, resulting in a total of 211 ABA-dependent and 1,118 ABA-independent drought-responsive genes. Functional analysis showed that ABA-dependent DEGs were significantly enriched in the expected biological processes responding to water deprivation (false discovery rate, FDR < 8.7x10^-12^ ) and ABA stimulus (FDR < 5.0x10^-8^; Figure 2A; a full list of enriched GO terms is shown in Table S2). On the other hand, the functional enrichment of ABA-independent DEGs revealed that these genes were preferentially enriched in responses to jasmonic acid (JA, FDR < 3.6x10^-13^), salicylic acid (SA, FDR < 3.2x10^-2^) and gibberellin stimuli (GA, FDR < 4.3x10^-2^; Figure 2B; a full list of enriched GO terms is shown in Table S3), indicating that ABA-independent genes were involved in several other hormone signaling pathways during the response to drought stress. Moreover, it was interesting that both ABA-dependent and ABA-independent DEGs tended to be involved in glycosinolate and glycoside biosynthetic processes.

#### Validation of differentially expressed genes by RT-qPCR

**Figure 3:**
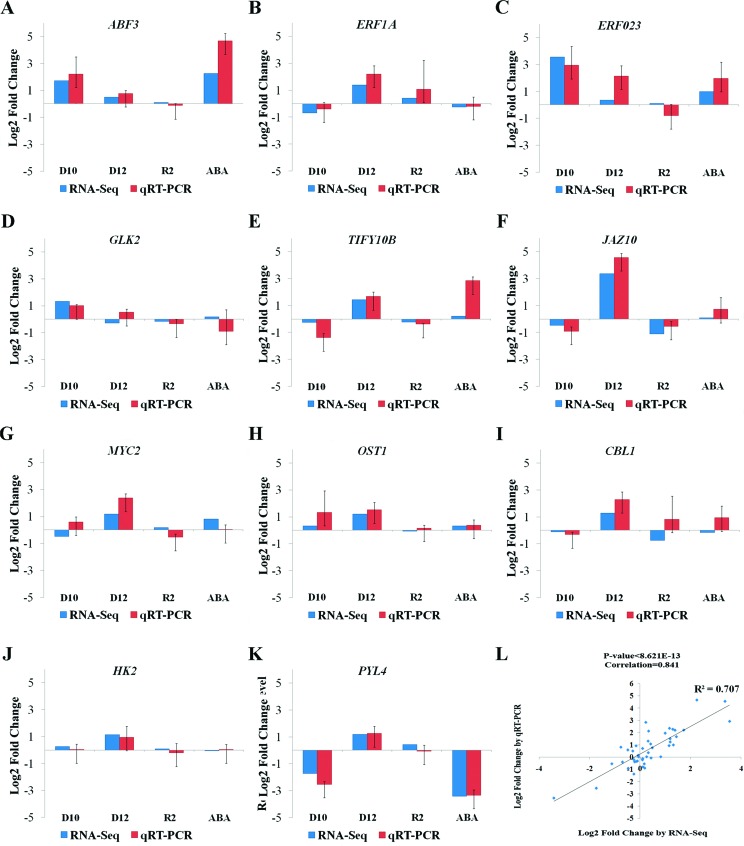
Quantitative RT-PCR validations of differentially expressed genes in four situations. (A) to (K) are the genes *ABF3* (AT4G34000), *GLK2* (AT5G44190), *ERF023* (AT1G01250), *ERF1A* (AT3G23240), *MYC2* (AT1G32640), *HK2* (AT5G35750), *CBL1* (AT4G17615), *OST1* (AT4G33950), *PYL4* (AT2G38310), *JAZ10* (AT5G13220) and *TIFY10B* (AT1G74950), respectively. The data are presented as the log2 value of the fold change from RT-qPCR and RNA-Seq. D10, D12, R2 and ABA represent plants under drought stress for 10 days, 12 days, rewatered for 2 days, and sprayed with 10 (μmol/L ABA for 4 hours, respectively. Error bars represent the mean ± SD of three biological replicates. (L) Pearson correlation analysis between the results of the two methods was performed, and a scatter plot was generated.

To further confirm the gene expression patterns observed in the RNA sequencing data, RT-qPCR analyses were performed on 11 genes, including *ABF3* (AT4G34000), *GLK2* (AT5G44190), *ERF023* (AT1G01250), *ERF1A* (AT4G17500), *MYC2* (AT1G32640), *HK2* (AT5G35750), *CBL1* (AT4G17615), *OST1* (AT4G33950), *PYL4* (AT2G38310), *JAZ10* (AT5G13220) and *TIFY10B* (AT1G74950). The expression patterns of most of the genes as determined by RNA sequencing was similar to that determined by RT-qPCR (Figure 3A-K). Furthermore, we used a Pearson’s correlation analysis to assess the agreement between the RNA sequencing and the RT-qPCR data. As shown in Figure 3L, most of the RNA sequencing results significantly correlated with the RT-qPCR results with a *p*-value < 8.6x10^-13^. These data indicated that the RNA sequencing data were in good agreement with the expression profiles determined by qRT-PCR analysis in this study.

#### Interaction network between ABA-dependent and ABA-independent drought-responsive genes

**Figure 4:**
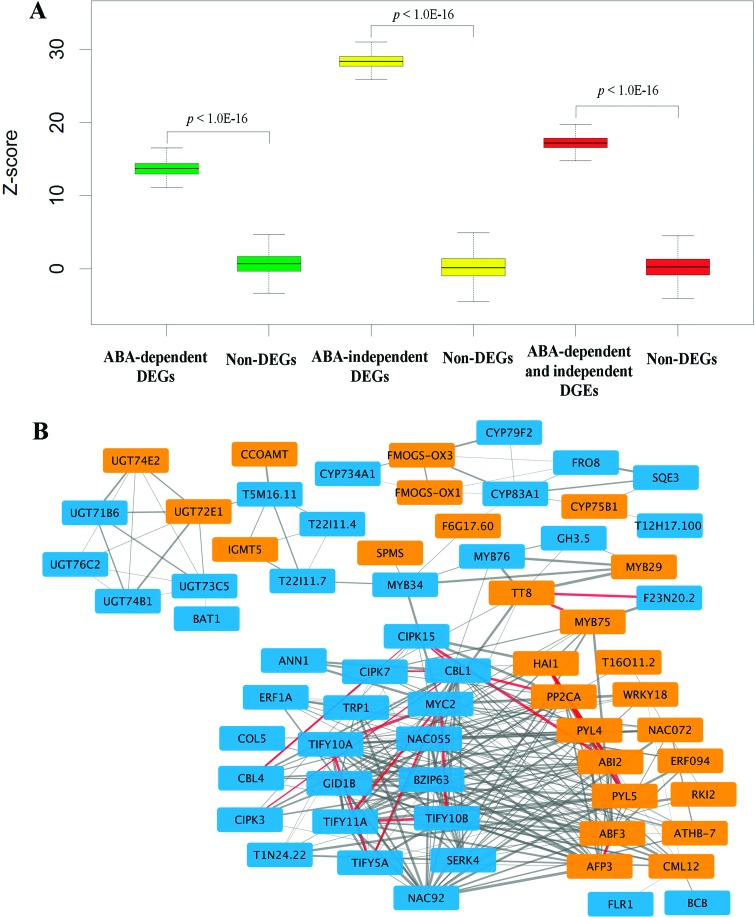
Interaction analysis between ABA-dependent and ABA-independent drought-responsive genes. (A) Statistical significance of the global interactions between ABA-dependent and ABA-independent drought-responsive genes. The significance of interactions among drought-responsive gene sets was calculated using t-tests by comparing the Z-scores of the interactions of DEGs and non-DEGs. The random gene sets were selected from the pool of Non-DEGs of the same size as the drought-responsive gene sets to calculate the Z-score. The boxplots indicate inter-quartile ranges of the data. The bar in each boxplot indicates the median. (B) Network view of interaction between ABA-dependent and ABA-independent drought-responsive genes. ABA-dependent DEGs are depicted in orange, and ABA-independent DEGs are depicted in blue. The interactions connecting the nodes are represented by color-coded lines according to the experimental verification status. Gray represents the predicted interaction without experimental verification; red represents the predicted interaction with experimental verification. The line width represents the probability of the protein pairs interacting.

We previously developed a genome-wide PPI network that encompasses 316,747 high-confidence interactions among 12,574 proteins (Zhang *et al.*, 2016). Based on the global protein interaction map, we investigated possible interactions among the identified DEGs to provide insight into the interplay of ABA-dependent and ABA-independent genes in response to drought stress. To evaluate the interaction confidence between ABA-dependent and ABA-independent gene sets, we compared the global interactions among DEGs with those of non-DEGs in AraPPINet using the Z-score method. We found that the interactions of drought-responsive genes, including the interactions within and between ABA-dependent DEGs and ABA-independent DEGs, all significantly exceeded that of non-DEGs of the same size in the AraPPINet network (Figure 4A). Furthermore, co-expression analysis based on the ATTED network was also carried out to investigate relationships of these DEGs (Aoki *et al.*, 2016). As shown in Figure S2A-C, there were significant co-expression relationships between ABA-dependent and ABA-independent DEGs with 2,915 significant links (the top 1% correlated gene pairs), approximately two-fold enrichment than non-DEGs. All these results suggest that ABA-dependent genes significantly interact with ABA-independent genes in response to drought stress.

Although some genes are involved in the interplay between ABA-dependent and ABA-independent signaling pathways (Roychoudhury *et al.*, 2013), the system interactions of these two pathways under drought stress is still unclear. To determine which drought-responsive genes play core roles in the interaction between ABA-dependent and ABA-independent pathways, we estimated the significance of each drought-responsive gene linked to genes from the other set. A total of 32 ABA-dependent genes were found to significantly interact with the ABA-independent gene sets based on the criteria of an empirical *p*-value < 0.05 (Table S4). Similarly, 62 ABA-independent genes were preferentially linked to genes from the other set (Table S5). All 94 nodes involving 282 potential interactions probably function in the crosstalk between ABA-dependent and ABA-independent signaling pathways in response to drought stress. The partial PPI network was presented using Cytoscape based on the ranking significance (Figure 4B). A topological structure analysis revealed that this high-confidence network was composed of three modules, one major and two small. For the major module, known ABA-dependent genes, including *PYL4, PYL5* (AT5G05440), *ABI2* (AT5G57050), *AHG3* (AT3G11410), *ABF3* and *RD26*(AT4G27410), and ABA-independent genes, such as *JAZ1* (AT1G19180), *JAZ5* (AT1G17380), *MYC2, NAC3* (AT3G15500), *NAC6* (AT5G39610), *CBL1* and *CIPK15* (AT5G01810), were involved in the response to drought stress. In the drought-responsive PPI network, some interactions have been experimentally verified, such as the interaction between AHG3 and CBL1 (Lan *et al.*, 2011) as well as the interaction between ABI2 and CIPK15 (Christmann *et al.*, 2006). These results suggest that a number of genes are core components involved in the interplay between ABA-dependent and ABA-independent pathways in response to drought stress.

#### Crosstalk model of ABA and other hormone signaling in drought response

**Figure 5:**
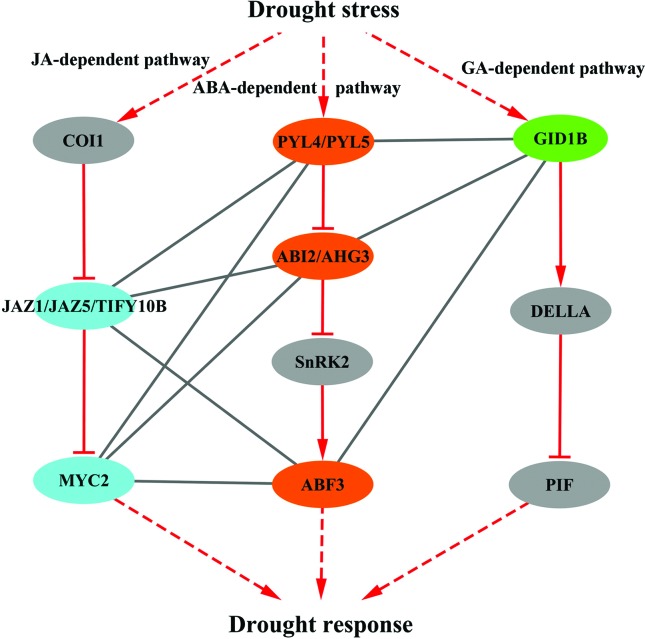
A model for the interplay of ABA and the other two hormone signaling pathways in response to drought stress. The components of three different hormone pathways are represented by three different colors. ABA-dependent drought-responsive genes are depicted in orange, and ABA-independent drought-responsive genes involved in JA and GA signaling are depicted in sky blue and green, respectively. Interactions among those components are indicated by line; the gray line indicates the predicted interaction, and the red line indicates that the relationship has been experimentally verified. The red dotted lines indicate multi-step routes.

The pathway mapping of the candidate interacting components in response to drought stress showed that six ABA-dependent genes were core components in ABA signaling and five ABA-independent genes were involved in the JA and GA signaling pathways (Kanehisa *et al.*, 2016). As shown in Figure 5, the proteins in the ABA signaling pathway, including ABA receptors (PYL4 and PYL5), PP2C proteins (ABI2 and AHG3), and bZIP transcription factor ABF3, were predicted to interact with JAZ proteins, including JAZ1, JAZ5 and TIFY10B, and the transcription factor MYC2. These are novel interactions that probably function in the interplay between ABA and JA signaling pathways in response to drought stress. On the other hand, five proteins (ABF3, PYL4, PYL5, ABI2 and AHG3) were also predicted to interact with the GA receptor GID1B from the GA pathway, suggesting possible crosstalk between ABA and GA signaling pathways under drought stress.

#### MYC2 can interact with ABF3

**Figure 6:**
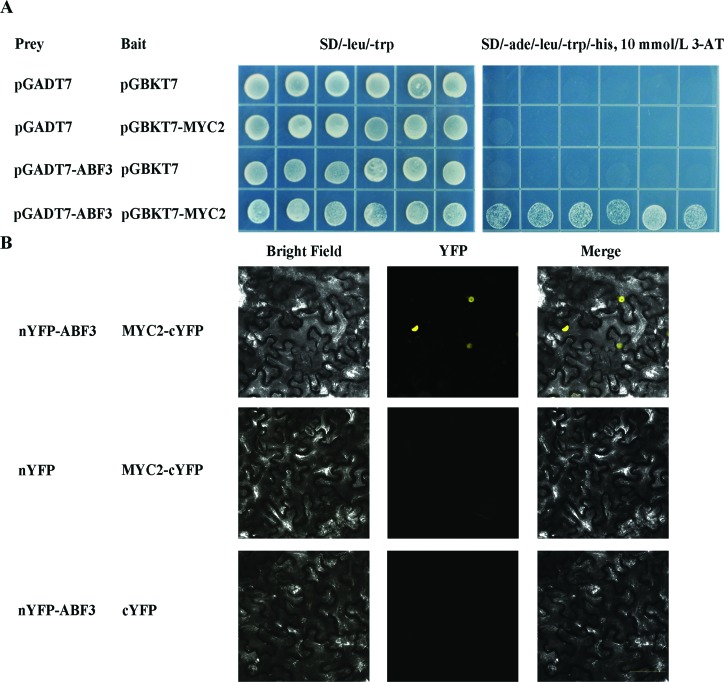
MYC2 interacting with ABF3 *in vivo.* (A) Yeast two-hybrid assays for interaction between MYC2 and ABF3. The combinations of pGADT7 with pGBKT7, pGADT7 with pGBKT7-MYC2, and pGADT7-ABF3 with pGBKT7 were used as controls. 10 mM 3-AT was added to inhibit self-activation on 4D plates. Three replicating experiments were conducted, and six different colonies were investigated for each type of combination. (B) BiFC assays for interaction between MYC2 and ABF3. Co-expressing nYFP-ABF3 and MYC2-cYFP, nYFP-ABF3 and cYFP, as well as MYC2-cYFP and nYFP in tobacco leaf epidermal cells. YFP signal intensity was detected from 48 h after infiltration. Scale bar: 50 μm.

According to the ranking results of interaction significance, ABF3 is a key component in ABA signaling, with the highest interaction significance between the JA and GA signaling pathways, suggesting that ABF3 is a convergence point in these pathways in response to drought stress. As an ABF3-interacting candidate, MYC2 has roles in both JA and ABA signaling (Abe *et al.*, 2003; Lorenzo *et al.*, 2004). It is possible that MYC2 might be involved in the regulation of ABA signaling by interacting with ABF3 under drought stress. To validate our predition about the interactions between ABA and JA signaling pathways, we used yeast two-hybrid assays to test the interactions between ABF3 and MYC2. As shown in Figure 6A, there is clearly a strong interaction between ABF3 and MYC2 in yeast.

To further elucidate the interaction between MYC2 and ABF3 in plant cells, we conducted a bimolecular fluorescence complementation (BiFC) assay. YFP fluorescence was detected at the combination of nYFP-ABF3 and MYC2-cYFP (Figure 6B). In contrast, no fluorescence signal appeared in controls, in which nYFP-ABF3 was co-expressed with cYFP only, and MYC2-cYFP was co-expressed with nYFP only (Figure 6B). The result indicates that MYC2 physically interacts with ABF3 in plant cells, connecting ABA and JA signaling pathways. These findings support the idea that the interaction between ABF3 and MYC2 may mediate the crosstalk between ABA and JA signaling in response to drought stress.

### Discussion

Drought is a major environmental stress factor that affects the growth and development of plants. Numerous genes are induced when plants respond to drought stress. In this study, we identified more than 1,600 genes that were significantly differentially expressed under prolonged drought stress, which is less than the number of genes significantly perturbed early in response to drought stress (Harb *et al.*, 2010). These drought-responsive genes are mainly involved in two types of pathways: ABA-dependent and ABA-independent pathways (Gosti *et al.*, 1995). The comparative transcriptome analysis of drought stress and ABA treatment revealed that more ABA-independent genes were involved in the response to prolonged moderate drought stress. More interestingly, we found that some genes in the two pathways play similar roles in drought-responsive processes. This was substantiated by a GO analysis, which showed enrichment for genes involved in cellular processes, such as the glycosinolate and glycoside biosynthetic processes. Glucosinolates belong to secondary metabolites in plant, and might play an important role in avoiding loss of water in plant through closing the stomata. The system of glucosinolate and myrosinase is necessary for crucial responses to ABA of guard cells (Zhao *et al.*, 2008). Thus, the water limitation and exogenous ABA treatment may have triggered an active interaction between the plant and the environment. In addition, the functional enrichment of ABA-independent drought stress genes revealed that they were preferentially enriched in other hormone pathways, including JA, GA and SA signaling, as shown in previous studies (Bari and Jones, 2009).

The crosstalk between different plant hormones is important for the plants’ response to abiotic stress (Peleg and Blumwald, 2011). Recent studies have raised the intriguing possibility of a crosstalk between ABA and other signaling pathways in response to drought stress (Yoshida *et al.*, 2015). Three proteins, ABI2, ABI1 and MYC2, play important roles in the crosstalk between the ABA signaling pathway and the ethylene signaling pathway in response to environmental stress (Atkinson and Urwin, 2012). As an ethylene-responsive transcription factor, SUB1A can mediate the interaction between these pathways in response to drought stress (Sharma *et al.*, 2013). Moreover, DELLA as a key component in GA signaling, likely mediating the interaction between the GA and ABA signaling pathways (Zentella *et al.*, 2007). This increasing molecular evidence suggests that ABA and other hormone signaling pathways are interwoven with each other into an intricate network to regulate plant responses to drought stress (Verma *et al.*, 2016).

To enhance our understanding of the interplay between ABA-dependent and ABA-independent stress signaling pathways, we used a network-based approach to explore the highly possible interactions functioning in the crosstalk between the two pathways in drought response. The comparative analysis of PPI and co-expression networks revealed significantly enriched interactions between ABA-dependent and ABA-independent drought-responsive genes, suggesting that the plant response to drought stress occurred through the interplay of ABA and other hormone signaling pathways. In addition, we identified 32 ABA-dependent and 62 ABA-independent genes as core components involving approximately 300 interactions probably functioning in the crosstalk between the two major signaling pathways under drought stress. Among these interacting genes, six known genes play important roles in ABA signaling, and five ABA-independent genes are key components involved in the JA and GA signaling pathways (Kanehisa *et al.*, 2016). More specifically, ABF3 was identified as an important protein for the inter-pathway communication of ABA with the JA and GA signaling pathways, suggesting that ABF3 probably served as a convergence point in these pathways under drought stress.

As another crucial transcription factors in the model, MYC2 is known to be a positive regulator in ABA signaling pathway (Abe *et al.*, 2003), and furthermore, it is characterized as a central regulator in JA signaling pathway (Lorenzo *et al.*, 2004). Recent studies have revealed MYC2 as a master regulator of both the ABA and JA signaling pathways, suggesting its involvement in the regulation of the crosstalk between the two pathways under drought stress (Kazan and Manners, 2013; Riemann *et al.*, 2015). Most recently, it has been shown that MYC2 can interact with ABA receptor PYL6, which provide a direct link between the two signaling pathways (Aleman *et al.*, 2016). In this study, MYC2 was predicted to interact with the ABA core transcription factor ABF3 based on our crosstalk model and was also validated by yeast two-hybrid assays.

These findings suggests that the crosstalk between ABA and JA signaling might be mediated through the interaction between MYC2 and ABF3 to regulate the expression of drought stress responsive genes. This verified interaction provided actual support to our prediction about the network among genes in ABA-dependent and ABA-independent pathways. Although most of predicted interactions still require experimental confirmation, the current picture provides a global map to help uncover the complex crosstalk between ABA and other hormone signaling pathways in drought response.

## Conclusions

In the present report, we identified 211 ABA-dependent genes and 1,118 ABA-independent genes involved in the drought stress response using an RNA Sequencing approach. By combining network analyses we found significantly enriched interactions between ABA-dependent and ABA-independent pathways, with 94 genes acting as core interacting components in response to drought stress. Furthermore, the direct interaction of ABF3 with MYC2 was validated by experimental assays. Our study provides a systematic view of the relationship between ABA-dependent and ABA-independent pathways and a reference to uncover novel interactions of ABA and other hormone signaling pathways under drought stress.

## Supplementary material

The following online material is available for this article:

Figure S1 - Number of genes detected by at least five mapped reads for each sample at different sequencing depth.

Figure S2 - Co-expression analysis between ABA-dependent and ABA-independent drought-responsive genes.

Table S1 - Primers used for qRT-PCR analysis.

Table S2 - Full list of enriched GO terms of ABA-dependent drought-responsive genes.

Table S3 - Full list of enriched GO terms of ABA-independent drought-responsive genes.

Table S4 - List of ABA-dependent genes that significantly interacted with ABA-independent genes.

Table S5 - List of ABA-independent genes that significantly interacted with ABA-dependent genes.
