# Supplementary material for: Network analysis of ABA-dependent and ABA-independent drought responsive genes in Arabidopsis thaliana
Source: Genet Mol Biol. 2018 Jul 23;41(3):624–37. doi: 10.1590/1678-4685-GMB-2017-0229 (PMC6136374; doi:10.1590/1678-4685-GMB-2017-0229)
Supplement: Supplementary file 5 [file 1415-4757-GMB-1678-4685-GMB-2017-0229-s002.pdf]

Supplementary Material to “Network analysis of ABA-dependent and ABA-independent drought responsive genes in *Arabidopsis thaliana*”

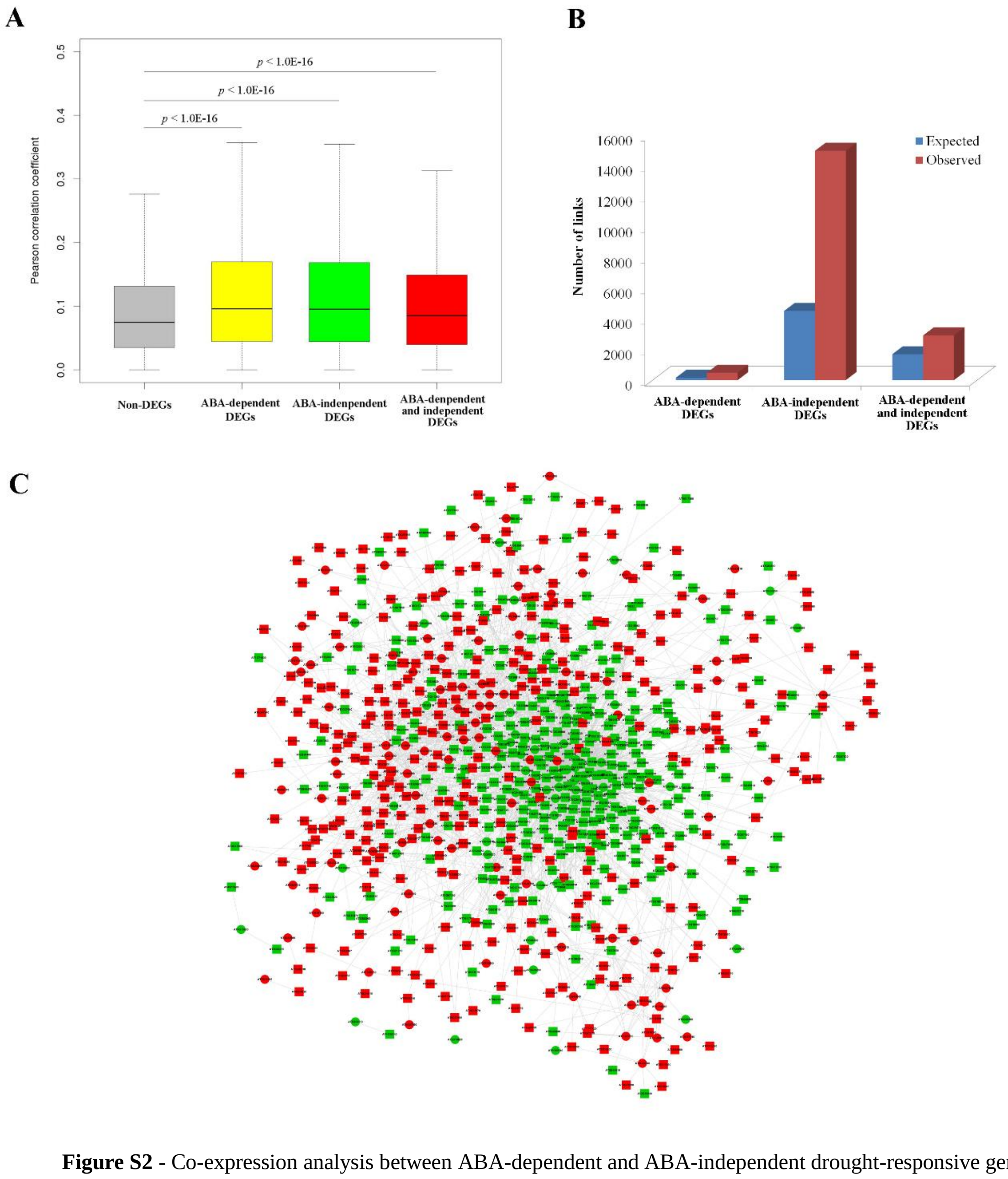

Figure S2 - Co-expression analysis between ABA-dependent and ABA-independent drought-responsive genes.
